# Prenatal Ultrasound Diagnosis of Biometric changes in the Brain of Growth Restricted Fetuses. A Systematic Review of Literature

**DOI:** 10.1055/s-0041-1730290

**Published:** 2021-08-30

**Authors:** Patrícia Isabel Pereira Silva, Miriam Perez

**Affiliations:** 1 Hospital Dr. Nélio Mendonça Funchal, Portugal

**Keywords:** fetal growth restriction, diagnostic imaging, brain injuries, ultrasonography, neurosonography, restrição de crescimento fetal, imagem de diagnostic, lesões cerebrais, ultrassonografia, neurosonografia

## Abstract

Fetal growth restriction (FGR) occurs when the fetus does not reach its intrauterine potential for growth and development as a result of compromise in placental function. It is a condition that affects 5 to 10% of pregnancies and is the second most common cause of perinatal morbidity and mortality. Children born with FGR are at risk of impaired neurological and cognitive development and cardiovascular or endocrine diseases in adulthood. The purpose of the present revision is to perform a literature search for evidence on the detection and assessment by ultrasound of brain injury linked to FGR during fetal life. Using a systematic approach and quantitative evaluation as study methodology, we reviewed ultrasound studies of the fetal brain structure of growth-restricted fetuses with objective quality measures. A total of eight studies were identified. High quality studies were identified for measurement of brain volumes; corpus callosum; brain fissure depth measurements, and cavum septi pellucidi width measurement. A low-quality study was available for transverse cerebellar diameter measurement in FGR. Further prospective randomized studies are needed to understand the changes that occur in the brain of fetuses with restricted growth, as well as their correlation with the changes in cognitive development observed.

## Resumo

A restrição do crescimento fetal (RCF) ocorre quando um feto não consegue atingir seu potencial de crescimento intrauterino, na maioria das vezes por compromisso da função placentária. É uma condição que afeta de 5 a 10% das gravidezes e é a segunda causa mais comum de morbidade e mortalidade perinatal. Crianças nascidas com RCF incorrem em maior risco de atraso no desenvolvimento neurológico e cognitivo, bem como de doenças cardiovasculares e/ou endócrinas, na idade adulta. O objetivo desta revisão foi o de pesquisar na literatura evidência sobre o diagnóstico pré-natal por ecografia de lesões cerebrais relacionadas com a RCF. Utilizando uma abordagem sistemática, avaliamos de forma quantitativa a metodologia dos oito estudos que preencheram os critérios de inclusão e foram, assim, incluídos nesta revisão. Foram identificados estudos de alta qualidade para a medição dos volumes cerebrais; medição do corpo caloso; medição da profundidade das incisuras cerebrais e medição do cavum do septo pelúcido. Os autores identificaram um estudo de qualidade inferior sobre a medição transversal do diâmetro transcerebelar em fetos com RCF. Mais estudos prospectivos randomizados são necessários para perceber quais as alterações que ocorrem no cerébro dos fetos com restrição do seu crescimento, bem como, a sua correlação com as alterações do desenvolvimento cognitivo observadas.

## Introduction

Fetal growth restriction (FGR) occurs when the fetus does not reach its intrauterine potential for growth and development as a result of compromise in placental function. It is a condition that affects 5 to 10% of pregnancies and is the second most common cause of perinatal morbidity and mortality. 1 The diagnosis of fetal “smallness” is currently performed on the basis of an estimated fetal weight (EFW) below a given threshold, most commonly the 10th percentile. 2 In 2014, Figueras et al., 2 introduced a new concept of early-onset and late-onset of FGR. Early-onset FGR is typically diagnosed in the second trimester, it is strongly associated with severe placental dysfunction and chronic fetal hypoxia, it presents with preeclampsia in up to 50% of cases and tends to describe the more severe cases of FGR. Late-onset FGR is the more common form, present in 70 to 80% of FGR, and typically becoming apparent in the 3 ^rd^ trimester (32 weeks) of pregnancy. 2 Evidence accumulating over the last 20 years has consistently demonstrated how being born small has important implications for the quality of health during adulthood, among which are impaired neurological and cognitive development and cardiovascular or endocrine diseases in adulthood. 3 4

Poor placental function is the most important contributor to FGR, resulting in chronic fetal hypoxia and hypoglycemia in an otherwise normal fetus. 5 In turn, chronic fetal hypoxemia and nutrient insufficiency directly decrease fetal growth rate, and hypoxia induces a redistribution of cardiac output. 6 This redistribution of fetal cardiac output tends to protect the brain growth relative to other organs, and is termed brain-sparing or central redistribution, but this does not ensure normal brain development. 7 Children with FGR born preterm or with evidence of brain-sparing are considered to be at greatest risk for deficits in brain maturation; 8 hence, this blood flow redistribution seems to favor some brain regions over others. So, contrary to what was thought, cardiac output redistribution is not necessarily effective in protecting the brain. 9 Both early and late-onset FGR fetuses with brain sparing effects have worse abnormal neurobehavior in the neonatal period and at 2 years of age. 10

Human FGR imaging studies and postmortem examination, together with animal experimental studies of placental insufficiency and FGR, describe reduced total brain volume, with loss of both gray and white matter substructure. 11 At the cellular level, gray matter areas are shown to have reduced cell number with sparse and disorganized cortical structure. 11

The complex and heterogeneous adverse outcomes observed in FGR children demonstrate the need for accurate neurological assessments that can be applied either antenatally or postnatally. Since FGR babies are often delivered early, brain injury could be either a result of prenatal insult or it could be a result of prematurity, or both. Prenatal brain studies in FGR are paramount, as postnatal series are incapable of differentiating prenatal vs postnatal injury.

The purpose of the present revision is to perform a literature search for evidence for the detection and assessment by ultrasound of brain injury linked to FGR in fetal life.

## Methods

The current systematic review was conducted in line with the Preferred Reporting Items for Systematic Reviews and Meta-Analyses (PRISMA) statement, a 27-item checklist for the reporting of systematic reviews and meta-analyses.

We conducted a literature search in June 2019 on the CINAHL, MEDLINE and PUBMED databases using a combination of indexed and free terms obtained based on the elements of the Population, Intervention, Comparison and Outcome (PICO) mnemonic.

**P** – brain lesions/brain damage in fetal growth restriction or intrauterine growth restriction

**I** – prenatal diagnosis; ultrasound; neurosonography (NSG).

**C** – Non applicable

**O** – neurodevelopment

The eligibility criteria were as follow:

- Inclusion criteria: brain ultrasound or neurosonographic studies of human singleton or multiple pregnancies complicated by fetal growth restriction in the second or third trimester of pregnancy; no time limit; no restrictions of study design or methodology; journal articles written in English.
- Exclusion criteria: studies involving complementary means of diagnosis other than ultrasound; studies involving the brains of fetuses affected with other pathologies or genetic syndromes or fetuses with a normal development or preterm fetuses; studies involving only neonate evaluation of fetuses with fetal growth restriction during pregnancy.

The search was carried out individually for each database.

A first search was performed on June 9 using the following query:

*Intrauterine growth retardation* OR *Fetal growth restriction* OR *Fetal diseases* ) AND ( *Fetal intracranial hemorrhage* OR Cerebral lesions OR

*Brain abnormalities* OR *Brain lesions* ) AND ( *Prenatal diagnosis* OR

*Ultrasonography* , *prenatal* ).

The authors obtained 120 articles (24 from CINAHL; 21 from MEDLINE, and 75 from PUBMED). The titles and abstracts of these articles were analyzed, and those that met the inclusion criteria were selected. Then, the authors proceeded to the analysis of the keywords used in the articles that met the inclusion criteria and so to the refinement of the selected terms and query.

Then, a second survey was carried out on June 29 using the following query:

( *Fetal Growth Retardation/diagnostic imaging* OR *Fetal Growth Retardation /pathology* OR *Fetal weight* OR *Fetal Growth Retardation / physiopathology* OR *Infant, Small for Gestational Age* ) AND ( *Fetal brain* OR *Anterior Commissure, Brain* OR *Hypoxia-Ischemia, Brain* OR *Brain injuries* OR *Brain damage* OR *Brain/abnormalities* OR *Brain Diseases/physiopathology* OR *Brain/pathology* ) AND ( *Ultrasonography, Doppler, Pulsed* / *methods* OR *Ultrasonography, Prenatal/methods* OR *Ultrasonography, Prenatal* OR *Neuroimaging* OR *Neurosonography* ).

**Fig. 1:**
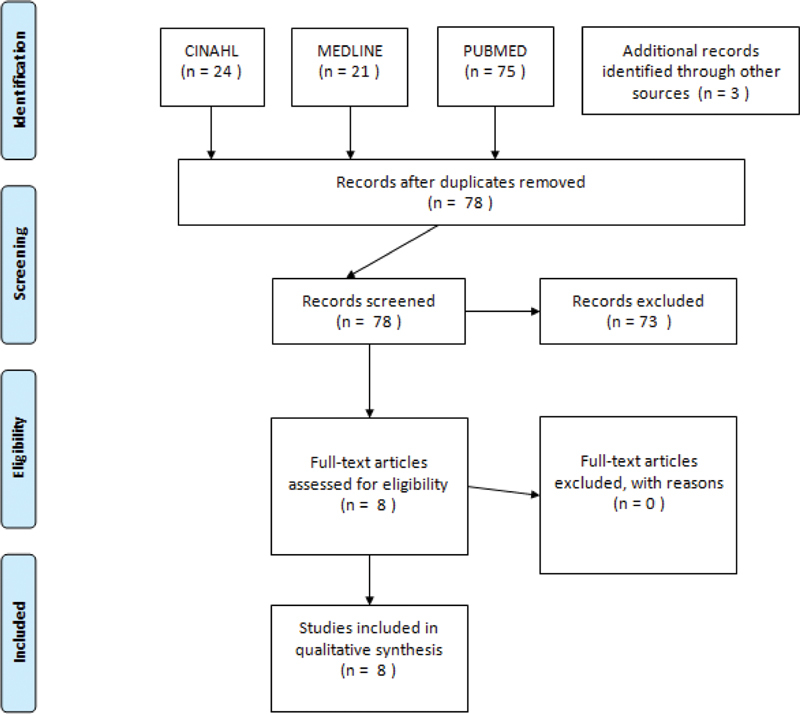
Consort flow diagram.

After removal of duplicates, the search generated 78 potential articles. We also used the ancestry approach, which involved searching the reference lists of review articles or articles dealing broadly with relevant subject matter, to uncover 3 additional potential papers. Titles and abstracts of the papers were assessed to determine whether the study was appropriate to be included in the current review, and, when this suggested an eligible study, the complete article was obtained. Full-text articles were retrieved and assessed based on the inclusion criteria for eligibility, eight of which were finally included ( Fig. 1 , Table 1 ). The main cause of exclusion of the articles were: postnatal evaluation of neonate brain following FGR during pregnancy; studies using magnetic resonance; studies that included brain evaluation of fetuses without FGR or fetuses with other pathologies or genetic syndromes; and studies that involved assessing neurodevelopment rather than assessing brain structures. The authors are aware of the sensitivity of MRI for the diagnosis of brain injuries, but also that this complementary mean of diagnosis is expensive, less accessible and implies compliance with certain rules for its performance. The inclusion of only ultrasound studies was thought and considered, and our aim was to understand the diagnostic scope of ultrasound, in particular NSG, to detect the changes in the FGR brain and, with this, try to create diagnostic protocols to be applied to this population.

**Table 1:** Studies included in qualitative synthesis

Citation	Study design	Brain structure	Restricted fetuses (N)	Doppler alterations	Weeks of gestation	Image study	Definition of fetal growth restriction
**Latif et al. (2017)** 12	Longitudinal	Total intracranial volume	68	UA and MCA*	32–36	3D – VOCAL**	EFW** < p10
**Benavides-Serralde et al. (2009)** 13	Cross-sectional	Total intracranial, frontal, thalamic and cerebelar volume	39	UA	28–34	3D – VOCAL**	EFW** < p10
**Caetano et al. (2015)** 14	Cross-sectional	Frontal, t otal intracranial and cerebelar volume	59	−	24–34	3D – VOCAL**	EFW** <p10
**Goldstein et al. (2011)** 15	Cross-sectional	Corpus callosum	24	−	16–36	2D	EFW** < p10
** Egaña-Ugrinovic et al. (2015) 16**	Longitudinal	Corpus callosum	98	CPR and UtA****	Third trimester	2D	EFW** < p10
**Husen et al. (2019)** 17	Longitudinal	Brain fissures	22	−	22, 26, and 32	3D	EFW** < p5
**Jacob et al. (2020)** 18	Cross-sectional	CSP, HC, TCD, LV and CM*****	247	−	?	2D	EFW** < p10
**Snijders et al. (1994)** 19	Cross-sectional	TCD	103	UA andUtA	19–39	2D	EFW** < p5

Abbreviations: *UA and MCA, umbilical artery and middle cerebral artery. **VOCAL, virtual organ computer-aided analysis. ***EFW, estimated fetal weight. **** CPR and UtA, Cerebroplacental ratio and uterine artery. *****CSP, HC, TCD, LV and CM, cavum septi pellucid, head circumference, transversal diameter of cerebellum, lateral ventricle and cisterna magna.

- Assessment of risk of bias

For assessment of risk of bias, the authors used a scoring system of methodological quality criteria ( Table 2 ) proposed by Ioannou et al. (2012). 20

**Table 2:** Methodological quality criteria proposed by Ioannou et al. (2012) 20

Domain	Low risk of bias	High risk of bias
** 1. Study design**		
1.1 Design	Clearly described as either cross-sectional or longitudinal	Not reported, mixture of both
1.2 Sample selection	Population-based study where there are attempts to identify and clearly define populations from a specific geographical area; from this underlying population, women are selected either consecutively or at random	Not population based; convenience sampling; arbitrary recruitment; or not reported
1.3 Number of occasions each fetus was measured (only for cross-sectional studies)	Each fetus was measured and included only once	Some fetuses were measured and included more than once
1.4 Method of selecting the gestational ages at which the fetuses were measured (only for longitudinal studies)	Interval of measures prospectively prespecified and justified	Interval of measures not prospectively prespecified and justified or not reported
1.5 Reason(s) for choosing a particular number of serial measurements (only for longitudinal studies)	Clear documentation of the intended number of serial measurements	No clear documentation of the intended number of serial measurements
1.6 Inclusion/exclusion criteria	The study made it clear that women at high risk of pregnancy complications were not included, and that women with abnormal outcome were excluded, i.e. an effort was made to include ‘normal’ outcome as best possible	The study population included both low-risk and high-risk pregnancies, or women with abnormal outcome were not excluded. Study population that did not exclude fetuses or women with the characteristics previously described. Exclusions which would have a direct effect on the estimated percentiles, such as fetuses found at birth to be large or small for dates.
1.7 Sample size	A priori determination/calculation of sample size and justification	Lack of a priori sample size determination/ calculation and justification
1.8 Data collection	Prospective study and ultrasound data collected specifically for the purpose of constructing charts of fetal size or fetal growth	Retrospective study, or data not collected specifically for the purpose of constructing charts of fetal size or fetal growth, or unclear (e.g. use of routinely collected data)
1.9 Method of dating pregnancy	Clearly described Known last menstrual period (LMP) and regular menstrual cycles prior to pregnancy AND a sonogram before 14 weeks demonstrating a crown–rump length (CRL) that corroborates.	Gestational age assessment at > 14 weeks, or gestational age assessment not including ultrasonographic verification
1.10 Collection of data on gestational age at inclusion	The gestational age was calculated precisely to the day	Truncation of gestational age to the number of ‘completed weeks’
** 2. Statistical methods**		
2.1 Number of measurements taken for each biometric variable	More than one measure per fetus per scan	Single measure or not specified
2.2 Statistical methods	Clearly described and identified	Not clearly described and identified
2.3 Assessment of increasing variability of the data with gestation	Performed	Not performed
2.4 Assessment of goodness of fit of the models	A test of goodness of fit of the models was reported	Goodness of fit of models was not reported
2.5 Scatter diagram of the data with the fitted percentiles superimposed	Study included scatter diagrams of the data with the percentiles superimposed	Study did not include scatter diagrams of the data with the percentiles superimposed
2.6 Change in reference percentiles across gestational age	Smooth change	Not smooth change
2.7 Methods used to estimate age specific reference intervals for fetal size measurements	Mean and standard deviation (SD) model', smoothed crude percentiles, or ‘LMS method	Inadequate
** 3. Reporting methods**		
3.1 Characteristics of study population	Presented in a table or clearly described, and includes minimum dataset of age, weight, height or body mass index and parity	Not presented in a table or not clearly described, or does not contain minimum dataset
3.2 Description of number approached/ enrolled	Described	Not described
3.3 Ultrasound machine(s) used	Clearly specified	Not clearly specified
3.4 Number of sonographers that took the measurements	Reported	Not reported
3.5 Description of measurement techniques	The study described sufficient and unambiguous details of the measurement techniques used for fetal size parameters, including imaging plane and caliper application method	The study did not describe sufficient and unambiguous details of the measurement techniques used for fetal size parameters
3.6 Contains quality control measures	Should include the following: – assessment of intraobserver variability – assessment of interobserver variability – image review – image scoring – image storage	Does not contain quality control measures
3.7 Report of mean and SD of each measurement and the sample size for each week of gestation	Presented in a table or clearly described	Not presented in a table or not clearly described
3.8 Report of regression equations for the mean (and SD if relevant) for each measurement	Reported	Not reported

This score is based on study design, statistical methods and reporting methods, to determine bias risk and ultimately, assign an overall quality score (percentage of low risk or bias marks over the total number of quality criteria). The rationale for adopting the checklist of Iaonnou et al. was the close parallel with the objectives of those authors to assess the methodological quality of studies in the field of fetal ultrasound involving measurements of fetal structures.

We present in tabular form the studies score with respect to design, statistical analysis, and reporting, together with the final computed score for methodological quality ( Table 3 ).

**Table 3:** Risk of bias in the included studies—Scoring through the methodological quality criteria proposed by Ioannou et al. (2012) 20

Domain	Latif et al. 12 Egypt 2017	Benavides-Serralde et al. 13 Spain/London 2009	Caetano et al. 14 Brazil 2014	Goldstein et al. 15 Israel 2011	Egaña-Ugrinovic et al. 16 Spain 2015	Husen et al. 17 The Netherlands 2019	Jacob et al. 18 Germany 2020	Snijders et al. 19 London 1994
1.1	High	High	Low	High	High	Low	High	High
1.2	Low	Low	Low	Low	Low	Low	Low	Low
1.3	High	Low	Low	Low	N/A	N/A	Low	Low
1.4	Low	N/A	N/A	N/A	Low	Low	N/A	N/A
1.5	Low	N/A	N/A	N/A	Low	High	N/A	N/A
1.6	Low	High	Low	Low	Low	Low	Low	Low
1.7	High	Low	Low	Low	High	Low	High	High
1.8	Low	Low	Low	High	Low	Low	High	Low
1.9	Low	High	Low	Low	Low	Low	Low	Low
1.10	Low	Low	Low	High	Low	High	High	Low
2.1	Low	Low	Low	Low	Low	High	Low	High
2.2	Low	Low	Low	High	Low	Low	Low	Low
2.3	Low	High	Low	High	Low	High	Low	High
2.4	Low	Low	Low	Low	Low	Low	Low	Low
2.5	Low	Low	Low	Low	Low	Low	Low	Low
2.6	High	High	Low	High	Low	Low	High	High
2.7	Low	Low	Low	Low	Low	Low	Low	Low
3.1	Low	Low	Low	High	Low	Low	High	High
3.2	Low	Low	Low	Low	Low	Low	Low	Low
3.3	Low	Low	Low	Low	Low	Low	Low	High
3.4	Low	Low	Low	Low	Low	Low	Low	High
3.5	High	Low	Low	Low	Low	Low	Low	High
3.6	High	Low	Low	Low	Low	Low	High	High
3.7	High	Low	Low	Low	High	Low	Low	High
3.8	Low	Low	Low	Low	Low	Low	Low	Low
**Quality score (%)**	**72**	**78**	**100**	**69**	**88**	**87**	**70**	**52**

Abbreviations: low = low risk of bias; high = high risk of bias, n/a = not applicable

## Results

The eight studies included came from different countries: Spain, London, Germany, The Netherlands, Egypt, and Brazil. Regarding the scientific impact of the articles, we can see that the article by Benavides-Serralde et al. (2009) 13 was cited 76 times, followed by the article by Snijders et al. (1994), 19 with 32 citations, Caetano et al. (2015), 14 with 10 citations, Goldstein et al. (2011), 15 with 8 citations, and, finally, Egaña-Ugrinovic et al. (2015) 16 was cited 6 times. 4 5 6 7 8 The authors present the synopsis of the results, providing the comparison and extraction of the key information of the different articles.

**Table 4:** Summary of the results of the articles included in the present revision

Citation	Objectives	N	Results
**Latif et al. (2017)** 12	To detect possible differences in fetal brain volumes between FGR, SGA fetuses with normal Doppler indices, and AGA fetuses.	68 FGR with brain-sparing effect 68 SGA 80 AGA	MCA-PI was significantly lower in FGR group compared with the other two groups. Brain volume was significantly lower in SGA and FGR groups compared with AGA.
**Benavides-Serralde et al. (2009)** 13	To evaluate the feasibility and reproducibility of volume segmentation of fetal intracranial structures using 3D ultrasound imaging, and to estimate differences in the volume of intracranial structures between intrauterine FGR and AGA fetuses.	39 FGR 39 AGA	All net volumes except those for the thalamus were significantly smaller in FGR fetuses. After adjusting volumes for biparietal diameter the frontal volume was significantly smaller and the thalamic volume significantly greater in FGR fetuses than in AGA fetuses. Significant intergroup differences in the ratios between structures were found only in those involving the frontal region.
**Caetano et al. (2015)** 14	To assess intracranial structure volumes by 3D sonography in fetuses with growth restriction	38 with EFW < 3rd percentile 21 with EFW between 3rd and 10th percentiles 54 controls	Statistical significance between the brain, frontal region, and cerebellar volumes and a relationship between the frontal region and the brain in fetuses with EFW below the 3rd percentile and controls were observed
**Goldstein et al. (2011)** 15	To characterize the normal ultrasonographic growth of the CC in normal and in growth-restricted fetuses throughout gestation.	252 AGA 24 FGR	A regression line of the CC was established through gestation and a second-degree correlation was found between gestational age and CC outer margin. In the growth-restricted fetuses the growth of the CC was significantly below both the 25th and 50th percentiles in 77.3% and 95.5%, respectively, for the same gestational age.
**Egaña-Ugrinovic et al. (2015)** 16	To explore CC developmental differences by neurosonography in late-onset small fetuses compared with AGA controls.	71 AGA 64 FGR 30 SGA	Small fetuses showed significantly shorter and smaller CC with smaller splenium compared to controls. The CC growth rate was also reduced when compared to controls. Changes were more prominent in small fetuses with abnormal cerebroplacental Doppler suggesting fetal growth restriction
**Husen et al. (2019)** 17	To examine differences in growth trajectories of fetal brain fissures in the FGR compared to controls.	22 FGR 172 AGA	Success rates for the Sylvian and insula depth measurements were over 80% and for POF over 62% at all GA. In FGR patients, compared to controls, the trajectory of the right Sylvian fissure depth was significantly decreased, while its growth rate was slightly increased, after adjustment for GA, head circumference, gender, educational level, and parity.
**Jacob et al. (2020)** 18	To assess whether fetal brain structures routinely measured during the second and third trimester ultrasound scans, particularly the width of the CSP, differ between fetuses SGA, fetuses VSGA and normal controls.	116 VSGA 131 SGA 136 AGA	The HC/CSP ratio differed notably between the controls and each of the other groups. No notable difference in the HC/CSP ratio between the VSGA and SGA groups could be found The HC/LV, HC/CM and HC/TCD ratios were similar in all the three groups.
**Snijders et al. (1994)** 19	To investigate TCD and TCD/AC in SGA fetuses thought to be growth-retarded due to uteroplacental insufficiency, and to relate the findings to other biometrical parameters, indices of fetal oxygenation, and pregnancy outcome.	103 FGR	Compared with the AC, HC, and FL, the TCD was relatively mildly reduced. However, in the 28 fetuses with TCD > 2 SDs below the normal mean, the degrees of growth retardation, acidemia, and erythroblastosis were more severe, and the incidence of perinatal death was higher than in the group with a normal sized TCD. Although in the group with TCD> 2 SDs below the normal mean the TCD/AC ratio was increased, in the most severely growth-retarded fetuses this ratio was usually within the normal range.

Abbreviations: AC, abdominal circumference; AGA, appropriate for gestational age; CC, corpus callosum; CM, cisterna magna; CSP, cavum septi pellucid; FGR, fetal growth restriction; GA, gestational age; HC, head circumference; LV, lateral ventricle; MCA-PI, middle cerebral artery pulsatility index; POF, parieto-occipital fissure; SD, standard deviation; SGA, small for gestational age; TCD, transverse cerebellar diameter; VSGA, very small for gestational age.

**Table 5:** Summary of absolute and relative findings for brain volumes

Citation	Absolute findings (cm ^3^ )					Relative findings			
**Latif et al.** 12	Brain volume	**SGA** 293.9 ± 11.9 ( *p* < 0.001) **FGR** 292.3 ± 12.4 ( *p* < 0.001) **AGA** 334.8 ± 19.4 ( *p* < 0.001)				HC is positively correlated with brain volume (r = 0.38, *p* = 0.001 and r = 0.76, *p* < 0.001, respectively)			
**Benavides-Serralde et al.** 13		**AGA**	**IUGR**		***P***		**AGA**	**IUGR**	***P***
	Total Intracranial Frontal Thalamic Cerebellar	194.2 ± 55.1 32.2 ± 11.6 1.5 ± 0.9 6.0 ± 2.1	157.3 ± 51.9 22.9 ± 9.9 1.3 ± 0.8 5.0 ± 1.7		0.001 0.001 0.23 0.001	Intracranial/thalamic Intracranial/cerebellar Intracranial/frontal Frontal/thalamic Frontal/cerebellar Thalamic/cerebellar	129.46 32.36 6.03 21.46 5.36 0.25	121.00 31.46 6.86 17.61 4.58 0.26	0.2 0.8 0.001 0.001 0.0122 0.289
**Caetano et al.** 14	**Mean**	**FGR** ***P*** ** < 3**	**FGR** **P 3–10**	**Control** **P 10–90**	***P***	**NA**			
	Brain volume	203.68	235.25	259.68	<. 001				
	Frontal region volume	97.42	113.63	130.74	<. 001				
	Cerebellar volume	7.22	8.13	9.21	.002				
	Frontal region/brain volume ratio	0.47	0.48	0.50	.008				

Abbreviations: AGA, appropriate for gestational age; FGR, fetal growth restriction; IUGR, intrauterine growth restriction; NA, non-applicable; *P* , *p* -value; SGA, small for gestational age.

**Table 6:** Summary of absolute and relative findings for brain fissures (Husen et al.) 17

		Model 1			Model 2			Model 3		
		β	95% CI	P	β	95% CI	P	β	95% CI	P
**Sylvian Left**	**FGR**	-2.788	-6.496; 0.919	0.140	-0.607	-4.423; 3.209	0.755	-0.544	-4.387; 3.299	0.781
	FGR * GA	0.004	-0.136; 0.023	0.627	0.001	-0.018; 0.020	0.913	0.001	-0.018; 0.019	0.948
**Sylvian Right**	**FGR**	-4.307	-7.894; -0.720	0.019	-4.296	-8.030; -0.562	0.024	-4.486	-8.182; -0.790	0.017
	FGR * GA	0.0128	-0.005; 0.030	0.151	0.019	0.001; 0.038	0.036	0.021	0.003; 0.039	0.025
**Insula Left**	**FGR**	-0.685	-4.142; 2.772	0.697	0.343	-3.296; 3.982	0.853	0.380	-3.282; 4.042	0.838
	FGR * GA	-0.002	-0.019; 0.015	0.796	-0.004	-0.022; 0.014	0.680	-0.004	-0.022; 0.014	0.688
**Insula Right**	**FGR**	0.744	-3.051; 4.539	0.700	1.944	-2.063; 5.952	0.341	1.981	-2.060; 6.023	0.336
	FGR * GA	-0.007	-0.026; 0.011	0.444	-0.008	-0.028; 0.011	0.411	-0.008	-0.028; 0.012	0.415
**POF Left**	**FGR**	-2.602	-7.469; 2.264	0.294	-0.392	-5.689; 4.904	0.884	-0.342	-5.680; 5.000	0.900
	FGR * GA	0.004	-0.020; 0.028	0.744	-0.001	-0.027; 0.025	0.954	-0.001	-0.027; 0.025	0.946
**POF Right**	**FGR**	0.247	-5.023; 5.517	0.927	3.357	-2.248; 8.961	0.240	3.171	-2.469; 8.811	0.270
	FGR * GA	-0.012	-0.038; 0.013	0.346	-0.022	-0.050; 0.006	0.117	-0.021	-0.049; 0.007	0.139

Abbreviations: FGR, fetal growth restriction; GA, gestational age. β, beta value; 95%CI, ninety-five percent confidence interval; *P* , *p* -value; POF, parieto-occipital fissure; HC, head circumference.

**Model 1** = Fissure = GA+ GA2 + covariate of interest.

**Model 2** = Multivariate: Fissure = GA + GA2 + Case + Case * GA + gender + education (low/middle) + parity + HC. **Model 3** = Model 2 + CPR

Model 1 represents the crude models investigating all covariates separately, model 2 is the multivariate model adjusted for educational level, parity, fetal gender and HC, model 3 is the multivariate model also adjusted for cerebro-placental ratio.

**Table 7:** Summary of absolute and relative findings for corpus callosum

Citation	Structure**	Late small fetusesP < 10	AGA	*p* *
**Egaña-Ugrinovic et al.** 16	Length/CI	0.493 ± 0.042	0.516 ± 0.052	< 0.01
	Anterior thickness/CI	0.061 ± 0.013	0.064 ± 0.014	0.25
	Middle thickness/CI	0.041 ± 0.007	0.043 ± 0.007	0.06
	Posterior thickness/CI	0.066 ± 0.016	0.068 ± 0.015	0.42
	Total area/CI	1.828 ± 0.432	2.034 ± 0.441	<0.01
	Rostrum area/CI	0.135 ± 0.054	0.186 ± 0.250	0.06
	Genu area/CI	0.283 ± 0.119	0.314 ± 0.110	0.09
	Rostral body area/CI	0.341 ± 0.076	0.367 ± 0.073	0.03
	Anterior midbody area/CI	0.224 ± 0.056	0.243 ± 0.051	0.03
	Posterior midbody area/CI	0.219 ± 0.06	0.235 ± 0.046	0.05
	Isthmus area/CI	0.206 ± 0.074	0.215 ± 0.055	0.40
	Splenium area/CI	0.473 ± 0.125	0.554 ± 0.185	< 0.01
**Goldstein et al.** 15	The CC growth was below the 25th percentile in 77.3% of the growth-restricted fetuses and below the 50th percentile in 95.5% of the growth-restricted fetuses for the same gestational age ( *p* < 0.00001)			

Abbreviations: AGA, appropriate for gestational age; CC; corpus callosum; CI; cephalic index; P, *p* -value.

** In order to correct the condition of a smaller head size influencing the size of brain structures in small fetuses, cephalic index (CI) was used as a normalization factor. Cephalic index was calculated using biparietal and occipitofrontal diameters by applying a previously reported formula: CI = BPD/OFD * 100.

**Table 8:** Summary of absolute and relative findings for cavum septi pellucid (Jacob et al.) 18

Absolute findings					Relative findings				
**Structure**	**VSGA**	**SGA**	**Controls**	***P*** **-value**	**Structure**	**VSGA**	**SGA**	**Controls**	***P*** **-value**
**HC, mm**	245.6	268.9	280.0	< 0.001	**HC/CSP**	48.2	47.7	51.3	0.022
**CM, mm**	6.3	6.6	6.8	0.063	**HC/LV**	53.0	55.4	54.5	0.459
**TCD, mm**	31.2	35.5	34.6	0.003	**HC/CM**	38.6	40.5	39.4	0.574
**LV, mm**	4.6	4.8	5.0	0.664	**HC/TCD**	7.7	7.6	7.9	0.137
**CSP, mm**	5.1	5.5	5.3	0.047					

**Latif et al. (2017)** 12 ***–Doppler and brain volumes measurements***

This study achieved a quality score of 72%. It is not clear whether it is a cross-sectional or longitudinal study. The study included 216 patients between 32 and 36 weeks of gestation, divided into 3 groups: group 1 included 80 appropriate for gestational age (AGA) fetuses, group 2 included 68 small for gestational age (SGA) with estimated fetal weights below the 10th percentile for gestational (GA) and normal Doppler index, and group 3 included 68 growth-restricted fetuses (FGR), with estimated fetal weights below the 10th percentile and abnormal umbilical artery (UA) Doppler; that is, pulsatility index (PI) > 95th percentile and/or absent or reversed end diastolic flow with brain-sparing effect (middle cerebral artery PI < 5th percentile). Routine two-dimensional (2D) ultrasound was used for fetal biometries, and Doppler measurements and three-dimensional (3D) ultrasound + virtual organ computer-aided analysis (VOCAL) mode were used for measurement of brain volume. Fetal biometry and 3D ultrasound measurements were done every 2 weeks in the 3 groups. Doppler measurements were done every 2 weeks in the AGA group, weekly in the SGA group, and twice weekly, or even daily when indicated, in the FGR group. All 3D and 2D ultrasound and Doppler measurements were performed with a 3.5 to 5 MHz abdominal probe using a Voluson E8 machine (GE Medical Systems, Zipf, Austria). Gestational age at ultrasound was not significantly different between the three groups. Despite 3D brain volume measurements being done every 2 weeks, there is no information about its evolution during pregnancy. The number of ultrasound operators and the inter or intraobserver variability were not described. The brain volumes were adjusted to the head circumference.

Doppler results : Umbilical artery PI were significantly higher in the FGR and the SGA groups compared with the AGA group. Middle cerebral artery (MCA) PI were significantly higher in the FGR and the SGA groups compared with the AGA group. CPR was significantly lower in the FGR group compared with the AGA and SGA groups, and it was also significantly lower in the SGA group compared with the AGA group ( *p* < 0.001).

Brain volume results : Brain volume was significantly lower in the SGA and FGR groups compared with the AGA group ( *p* < 0.001). No significant difference in brain volume was found between the SGA and FGR groups. In the SGA and FGR groups, head circumference (HC) was positively correlated with brain volume.

**Benavides-Serralde et al. (2009)** 13 **–Total intracranial, frontal, thalamic and cerebellar volumes measurements**

This study achieved a quality score of 78%. It is not clear in the text whether it is a cross-sectional or longitudinal study. The study included 77 patients between 28 and 34 weeks of gestation, divided into 2 groups: 39 AGA fetuses and 39 FGR fetuses matched by gestational age (±1 week). Fetal growth restriction was defined as an estimated fetal weight (EFW) < 10th percentile according to local standards and an UA (umbilical artery)-PI > 95th percentile.

Routine two-dimensional (2D) ultrasound was used for fetal biometries, and Doppler measurements and three-dimensional (3D) ultrasound and three-dimensional (3D) ultrasound + virtual organ computer-aided analysis (VOCAL) mode were used for measurement of brain volume. The brain volumes were adjusted to the biparietal diameter (BPD).

All ultrasound examinations were performed using a Voluson 730 Expert (GE Medical Systems) ultrasound machine with a 4 to 8 MHz curvilinear probe and an internal device for automatic acquisition of frames for volume reconstruction. Brain volumes were obtained by trained operators and were stored on digital devices for further analysis. The interobserver and intraobserver reliabilities were assessed by a two-way random and a two-way mixed model, respectively. There is no information on the gestational age at which the images were obtained.

Brain volume results : total intracranial, frontal, and cerebellar regions were significantly reduced in the FGR group ( *p* < 0.001). No statistically significant differences were found in the thalamic volume between the two groups.

Concerning the ratios between structures, in FGR fetuses the frontal volume was reduced, and the thalamic volume was increased, in relation to the total intracranial volume, but statistically significant differences were found only in ratios including the frontal volume (intracranial/frontal, frontal/thalamic and frontal/cerebellar).

After adjustment for BPD, the thalamic volume was found to be significantly larger, and the frontal volume significantly smaller, in FGR fetuses.

Substantial to almost perfect intraobserver reliability was observed for all regions. The only structure showing moderate interobserver measurement reliability was the thalamus.

**Caetano et al. (2015)** 14 **–Brain, frontal, and cerebellar volumes measurements**

This study achieved a quality score of 100%. The study included 77 patients between 24 and 34 weeks of gestation, divided into 2 groups: 54 AGA fetuses and 59 FGR fetuses (38 fetuses with EFW < 3rd percentile and 21 fetuses with EFW between the 3rd and 10th percentiles). Fetuses with alterations in the Doppler examination of the umbilical arteries (PI > 95th percentile for gestational age) were excluded.

Routine two-dimensional (2D) ultrasound was used for fetal biometries, and Doppler measurements and three-dimensional (3D) ultrasound and three-dimensional (3D) ultrasound + virtual organ computer-aided analysis (VOCAL) mode were used for measurement of brain volume. The brain volumes were not adjusted to the biparietal diameter (BPD) or head circumference.

All examinations were conducted with a Samsung Accuvix V20 (Samsung Medison Co. Ltd., Seoul, Korea) device equipped with a convex volume 4 to 7 MHz transducer. A single examiner performed the examinations; the examiner had 3 years of experience in 3D sonographic analysis in obstetrics.

The interobserver and intraobserver reliabilities were assessed by intraclass correlation coefficient (ICC). There is no information on the gestational age at which the images were obtained.

### Brain Volumes Results

There was a statistically significant difference in the brain volume between the group with weight predictions below the 3rd percentile and controls ( *p* < 0.001). Comparison of the group with weight predictions between the 3rd and 10th percentiles with controls showed no statistically significant difference.

For the frontal region volume , there was a statistically significant difference between the groups with weight predictions below the 3rd percentile and in the 3rd and 10th percentiles and controls ( *p* < 0.001). No statistically significant difference among growth-restricted groups was observed.

For the cerebellar volume , a statistically significant difference was only observed between the group with weight predictions below the 3rd percentile and controls. No statistically significant difference was observed among the growth-restricted groups and between the group with weight predictions between the 3rd and 10th percentiles and controls.

For the frontal region volume/brain volume ratio, a statistically significant difference was observed between the group with weight predictions below the 3rd percentile and controls, which showed a lower ratio and, thus, a lower frontal region volume when compared with the brain volume of the group with weight predictions below the 3rd percentile.

**Goldstein et al. (2011)** 15 **–corpus callosum measurement**

This study achieved a quality score of 69%. The study included 252 AGA and 24 FGR (EFW below the 10th percentile) fetuses between 16 and 36 weeks of gestation, with the purpose of evaluating the growth of the corpus callosum (CC) in both groups, throughout pregnancy.

Routine two-dimensional (2D) ultrasound was used for fetal biometries and for CC evaluation, which was performed in the sagittal plane. After identification of the entire CC, subsequent measurement of its longitudinal length, including the upper and the lower margins. The width between the upper and the lower curves was measured at the level of the body of the CC.

All examinations were conducted with Voluson Expert 730 (GE Medical Systems), Voluson Pro (GE Medical Systems), and Philips HDI 4000 (Philips Medical Systems, Best, Netherlands) ultrasound machines and by the same sonographer. The intraobserver reliability was assessed.

CC measurements results

- AGA fetuses : The length of the CC increased during pregnancy from 11 mm at 17 weeks' gestation to 39.7 mm at 33 weeks of gestation, a regression line of the CC was established through gestation and a second-degree correlation was found between gestational age and CC outer margin. The average growth in normal fetuses was 0.18 cm/week between 19 to 35 weeks of gestation.
- FGR fetuses: CC growth was below the 25th percentile in 77.3% of the growth-restricted fetuses and below the 50th percentile in 95.5% of the growth-restricted fetuses for the same gestational age ( *p* < 0.00001). Corpus callosum growth was also a discriminating feature between normal and growth-restricted fetuses. The average growth in FGR fetuses was 0.16 cm/week.

**Egaña-Ugrinovic et al. (2015)** 16 **–corpus callosum measurement**

This study achieved a quality score of 88%. It is not clear in the text whether it is a cross-sectional or longitudinal study. This study is part of a larger prospective research program on intrauterine growth restriction involving fetal, neonatal, and long-term postnatal follow-up.

The study included 71 AGA and 94 late-onset small fetuses (EFW < 10th percentile). These were subdivided in 64 FGR and 30 SGA, based on poor perinatal outcome factors, that is, birth weight < 3rd percentile and/or abnormal cerebroplacental ratio and/or uterine artery Doppler. The entire cohort was scanned to assess CC by transvaginal NSG obtaining axial, coronal, and midsagittal images. Corpus callosum length, thickness, total area, and the areas after a subdivision in 7 portions were evaluated by semiautomatic software. Furthermore, the weekly average growth of the CC in each study group was calculated and compared. The fetuses were followed-up from diagnosis in the 3rd trimester until delivery.

Routine two-dimensional (2D) ultrasound was used for fetal biometries and Doppler evaluation. Neurosonography was performed in both cases and controls using a two-dimensional transabdominal and transvaginal approach. The same equipment was used for all scans (Voluson 730 Expert scanner, equipped with a 5 to 9 MHz transvaginal transducer—GE Medical Systems). All NSGs were performed during the 3rd trimester of pregnancy and by 2 expert examiners. The CC measurements were adjusted to cephalic index.

Reliability between measurements from two observers blinded to group membership was assessed by the ICC.

### CC Measurements Results

Small fetuses showed a significantly reduced CC length and total CC area. Likewise, all the subdivisions of the CC had smaller areas, with significant differences in the rostral body, anterior midbody, and the splenium. After adjusting for potential confounding covariates, CC length, total CC area, and splenium remained significantly different between the two groups.

Morphometric comparison of the CC was then performed dividing small fetuses into FGR and SGA, as defined earlier. There was a significant linear trend across the study groups for shorter CC length, smaller total CC area, and smaller splenium. However, SGA fetuses had, in general, smaller corrected values as compared with controls. Concerning the growth, the average total CC area growth was lower in small fetuses compared to AGA (0.025/week vs. 0.035/week). Splenium growth (0.010/week vs. 0.027/week) was also lower in small fetuses.

**Husen et al. (2019)** 17 **–brain fissure depth measurements**

This study achieved a quality score of 87%. This study is part of a larger prospective research from the Rotterdam periconceptional cohort (predict study), an ongoing prospective cohort study with follow-up until birth.

The study included 172 AGA and 22 FGR (EFW below the 5th percentile) who were scanned at 22, 26, and 32 weeks of GA for 3D-ultrasound examinations of the fetal brain. The left and right sylvian, insula and parieto-occipital fissures (POF) were measured in standardized planes. Linear mixed models with adjustment for potential confounders were applied to estimate differences between the trajectories of brain fissure depth measurements of FGR and controls.

Routine 2D ultrasound was used for fetal biometries and Doppler evaluation and 3D ultrasound for brain fissure depth measurements. All tests were performed on the Voluson E8 system using a 1 to 7 MHz transabdominal transducer or a 6 to 12 MHz transvaginal transducer. A certified ultrasonographer carried out all ultrasounds, and *a posteriori* measurements were performed by one observer. The observer was blinded to the fetal group when identifying the fissures. The intraobserver reliability was acessed. The depths of brain fissures measurements were adjusted to potential confounders.

### Brain Fissure Depth Measurements

The growth trajectory of the right Sylvian fissure showed a significantly negative association with FGR fetuses compared with controls. Adjustment for GA, HC, gender, educational level, and parity showed comparable results, while the growth rate in millimeters per day of the right Sylvian fissure was slightly increased in FGR compared with controls.

No significant associations were found between FGR fetuses and the growth trajectories of the insula, POF, and left Sylvian fissure.

Significantly positive associations were shown between the HC and all brain fissures in the crude and in the fully adjusted model.

**Jacob et al. (2020)** 18 **–cavum septi pellucid width measurement**

This study achieved a quality score of 70%. It is a retrospective study that included the evaluation of archived sonographic scans of 116 very small for gestational age (VSGA) (EFW between the 3 ^rd^ and the 10 ^th^ percentile) fetuses, 131 SGA (EFW < 10th percentile) fetuses, and 136 normal controls. The following parameters were extracted from the VIEWPOINT database (Viewpoint Bildverarbeitung GmbH, Webling, Germany): GA, EFW, width of the cisterna magna (CM), transverse cerebellar diameter of the cerebellum (TCD), lateral ventricle (LV), the PI of the UA and the MCA as well as EFW percentiles. Lack of sufficient sonographic images rendered measurements unavailable in certain pregnancies (CM, TCD, LV, HC).

Cavum septi pellucid, EFW, GA and percentiles were determinable for all evaluated pregnancies. Axial views of the head were extracted from archived sonographic scans to determine the CSP width. One observer measured the CSP in its center, perpendicular to the brain's axis, placing the calipers on the inside of its lateral borders, as described by Abele et al. (cited by Jacob et al.) 18 In order to obtain parameters independent of the actual size of the fetus, the quotients HC/CSP, were calculated.

Results : There were statistically significative differences in the HC/CSP ratio between the SGA group and the control group as well as between the VSGA group and the control group. However, no distinction could be made between the SGA and the VSGA groups. The difference in the HC/CSP ratio shows that the CSP relative to HC is larger in VSGA and SGA fetuses than in the control population. However, the pairwise testing shows that there is only a remarkable difference between the control group and each of the other groups (SGA *P* = 0.017, VSGA *P* = 0.018); a *P* -value = 0.960 was used to compare VSGA and SGA. The GA during the ultrasound examination varied between 22 and 41 weeks and was not distributed evenly within the 3 groups.

**Snijders et al. (1994)** 19 **–fetal transverse cerebellar diameter measurements**

This study achieved a quality score of 52%. It is not clear in the text whether it is a cross-sectional or longitudinal study. The study included 103 SGA fetuses presumed to be growth-retarded due to uteroplacental insufficiency. The diagnosis of FGR was performed with fetal AC and, subsequently, birth weight below the 5th percentile of the appropriate reference range for gestation; the presence of an early diastolic notch in the waveform from at least one of the uterine arteries, and/or the absence of end diastolic frequencies in the waveform from the UAs. In this group of fetuses, the TCD, abdominal circumference (AC), femur length (FL), and HC were measured, and the TCD/AC, HC/AC and AC/FL ratios were calculated. The gestational age was 19 to 39 (mean = 31) weeks. In all cases, umbilical venous blood samples were obtained by cordocentesis.

Results : The mean TCD and umbilical venous blood pH were significantly below the appropriate normal mean for gestation, and the mean HC/AC, TCD/AC, and erythroblast count were increased.

In the 28 fetuses with TCD > 2 SDs below the normal mean, the FL, HC, AC, blood pH, and birth weight were lower, and the erythroblast count was higher than in the fetuses with a TCD within the normal range.

## Discussion

Fetal growth restriction is associated with an increased risk for neurodevelopmental impairment, with the degree of impairment related to the severity of growth restriction, the onset (early or late), and gestational age at birth (preterm or term). 7 We identified 8 studies on the ultrasound diagnosis of changes that may occur in fetal life in the brain of fetuses with growth restriction.

These studies, most of them case-control cross-sectional, covered different areas of the brain as well as used different diagnostic methodologies: 3D ultrasound for volume acquisition and depth measurement, 2D ultrasound +/− neurosonography with measurement of the length and width of several brain structures.

Since the purpose of this review was prenatal diagnosis by ultrasound, studies that included evaluation using magnetic resonance imaging were excluded and with them probable information about the pathophysiology of the impact of FGR on the fetal brain.

The authors chose to organize the discussion of the results grouping the studies with similar methodology / brain region, as follows: brain volumes measurements (3); corpus callosum measurements (2); brain fissure depth measurements (1); cavum septi pellucid width measurement (1) and fetal transverse cerebellar diameter measurement (1).

### Brain Volume Measurements

This group include 3 studies with similar methodology: Latif et al. (2017) 12 ; Benavides-Serralde et al. (2009) 13 and Caetano et al. (2015). 14 The highest quality score according to the checklist proposed by Ioannou et al. (2012) 20 was achieved by Caetano et al. 14

The 3 articles use 3D ultrasound using extended imaging virtual organ computer-aided analysis (VOCAL) method to calculate brain structural volumes of FGR fetuses in comparison with AGA fetuses.

Latif et al. 12 studied whole brain volume FGR with abnormal UA or MCA Doppler, Benavides-Serralde et al. 13 studied total intracranial, frontal, thalamic and cerebellar volumes in FGR fetuses with UA Doppler alterations and, finally, Caetano et al. 14 studied brain, frontal, and cerebellar volumes in FGR with normal Doppler values. All studies where consistent in demonstrating a reduced brain volume in FGR fetuses regardless of Doppler values, this was more evident in fetuses below the 3rd percentile, but also evident in SGA fetuses comparing with AGA.

Latif et al. 12 showed that the brain volume was significantly smaller in FGR group compared with AGA group ( *p* < 0.001), while no significant difference was found in brain volume between FGR group and SGA group. It seems that the decrease in brain volumes in SGA group despite that Doppler indices were within normal ranges but significantly different from AGA group, corroborate the hypothesis that redistribution of blood flow seems to be a sign of potential threat to the fetal brain.

The results of the study by Benavides-Serralde et al. 13 suggest that fetuses with severe intrauterine growth restriction have reduced frontal, total intracranial and cerebellar and increased thalamic volumes in relation to the total intracranial volume. These differences persisted when the volumes were adjusted by the biparietal diameter (BPD) of each fetus. The relative increase in thalamic volume in relation to other intracranial structures in FGR fetuses, already demonstrated in previous studies, 21 suggests that intrauterine growth restriction affects mainly the cortical white matter rather than the subcortical gray matter. 10

Early brain insults often lead to extensive neural reorganization of the gray and white brain matter, which can be expressed as an increment or reduction in specific brain areas, 22 in combination, hemodynamic brain vasodilatory response to hypoxia could be pathophysiological mechanism behind regional reorganization of the brain. 23 Whereas FGR fetuses at early stages of deterioration show an overall increment in blood flow perfusion, mainly manifested in the frontal lobe, those at later stages shift this increment to the basal ganglia. 13 The results of this study suggest that the fetal brain, exposed to a specific injury, does not respond in the same manner in all of its regions. It should be considered as a dynamic structure, which varies in its response depending on the onset, duration, and intensity of the injury. It would have been important to divide early-onset and late-onset in order to understand which brain changes are prevalent in each group.

Caetano et al. 14 *a* lthough evaluated fetuses with normal umbilical artery Doppler findings, showed similar results to those of Benavides-Serralde et al., 13 in which greater damage detected in the frontal region.

Regarding neurologic development, it used to be believed that the fetal brain would be protected, even in cases of UA Doppler alteration, by the mechanism of brain flow redistribution (“brain sparing effect”) These findings thus not corroborate the results of recent studies that have demonstrated an increased neurologic risk, regardless of umbilical artery Doppler alterations. 14

Other important feature demonstrated both in Benavides-Serralde et al. 13 and Caetano et al. 14 is a tendency for the frontal region to be more affected than other, this was evident both in fetuses with a EFW < 3rd percentile and in those with EFW between the 3rd and 10th percentiles. Regarding the frontal region of the brain, which is composed mostly of the frontal lobe, this region encompasses important neurologic areas, such as those for motor skills, language, behavior, personality, and thought. In addition to the functional importance of this region, previous studies have also demonstrated damage to the frontal cortex in cases of FGR, with decreased size and microstructural alterations diagnosed by magnetic resonance imaging. 24

Irrespective of quality score, these studies yielded valuable information about important findings in the brain of FGR fetuses and left several research hypotheses open.

### Corpus Callosum Measurements

This group includes 2 studies: Goldstein et al. (2011) 15 and Egaña-Ugrinovic et al. (2015). 16 The highest quality score according to the checklist proposed by Ioannou et al. (2012) 20 was achieved by Egaña-Ugrinovic et al. 16

Goldstein et al. 15 aimed to characterize the ultrasonographic growth of the CC in normal and in growth-restricted fetuses throughout gestation, and they did so in 252 AGA and 24 FGR, whereas Egaña-Ugrinovic et al. 16 intended to explore CC developmental differences by neurosonography in 64 late-onset FGR fetuses, 30 SGA and 71 normal controls.

Goldstein et al. 15 demonstrated that the length of the CC increases during pregnancy from nearly 11 mm at 17 weeks of gestation to 39.7 mm at 33 weeks of gestation. In symmetrical, growth-restricted fetuses, the CC growth was slower when compared to the normal-growth fetuses.

The length of the CC is likely to be affected by any reduction in white matter tracts during the preterm growth phase, in which the increasing bulk of the CC is in part due to development of the posterior third of the body of the CC (auditory fibers) and the splenium (visual fibers). 25 The CC originates at 10 to 11 weeks of gestation and first develops the rostrum to form the genu. The later development of the splenium and posterior area of the CC may explain why they appear particularly susceptible to damage in the second and third trimesters and in the perinatal period. 26

Egaña-Ugrinovic et al. 16 showed how small fetuses present differences in the linear and area measurements of the CC and have lower callosal growth rate assessed by NSG. These findings support those exposed by Goldstein et al., 15 and the notion that brain reorganization may affect white matter development in growth restricted fetuses 16 as well as that NSG can be a valid tool to detect such differences. In line with the results from Goldstein et al., 15 these authors also find that posterior portions of the CC were particularly affected with more marked changes in areas such as the splenium.

Although, late-onset IUGR fetuses with the presence of severity signs showed more accentuated shorter and smaller CC areas, in this study, the SGA group showed a trend for CC differences as compared to controls, which is line with the results mentioned above. Fetuses defined as SGA might suffer forms of growth restriction with impact in brain development that remain to be better characterized.

### Brain Fissure Depth Measurements

This cohort presented by Husen et al. (2019) 17 aimed to examine differences in the growth trajectories of fetal brain fissures in 22 FGR and 172 AGA fetuses by 3D ultrasound brain evaluation at 22, 26, and 32 weeks of gestation.

They found that the growth rate of the right Sylvian fissure was slightly increased in FGR compared to controls in the fully adjusted model (adjustment for GA, HC, gender, educational level, and parity). No significant differences in the trajectories of the left Sylvian, the insula, and POF were found between FGR fetuses and controls.

This result was not supported by other authors, 16 which may be explained by the diversity of the reported findings and the wide variety of measures and methods used to evaluate fetal brain development.

Although promising to enable evaluation of the brain fissures and, hence, brain development by ultrasound, this technique has limitations, such as the amount of cerebrospinal fluid that may influence the obtained brain fissure depth measurements and the different positions adopted by the fetus, which might lead to a slight deformation of the skull and, consequently, result in different fissure depth measurements.

The difference we find between FGR and controls is only seen in the trajectory of the right Sylvian fissure and not in the other fissures investigated in this study population. Whether we could interpret this finding as a delay or a disturbance remains unanswered. Larger studies and follow-up studies are necessary to further investigate whether or not other fissures are involved.

### Cavum Septi Pellucid Width Measurement

This retrospective study aimed to assess whether fetal brain structures routinely measured during the 2nd and 3rd trimester ultrasound scans, particularly the width of the CSP, differ among 131 SGA, 116 VSGA, and 136 AGA. The quality score of this study was 70%. The authors also intended to evaluate the transverse cerebellar diameter (TCD), the left ventricle (LV) and cistern magna (CM), but the lack of sufficient sonographic images rendered measurements unavailable in certain pregnancies. Cavum septi pellucid, EFW, GA, and percentiles were determinable for all evaluated pregnancies.

The results of this study show that the CSP, when set in relation to the HC (HC/CSP ratio), is notably larger in SGA and VSGA fetuses than in the control group.

There are certain limitations in this study, mainly due to its retrospective design, such as measurement deviations due to varying ultrasound planes by different examiners and inability to follow up on further development of the CSP pre- or postnatally.

### Fetal Transverse Cerebellar Diameter Measurement

This study presented by Snijders et al. (1994) 19 aimed to investigate TCD and TCD/AC in 103 SGA fetuses thought to be growth-retarded due to uteroplacental insufficiency and to relate the findings regarding other biometrical parameters, indices of fetal oxygenation, and pregnancy outcome. This article scored 53% in the methodological quality criteria scoring system proposed by Ioannou et al. 20 It is an older article, and it lacks some items, especially in the reporting methods section. In the 103 SGA fetuses, the mean TCD, AC, HC, FL, and umbilical venous blood pH were significantly below the appropriate normal mean for gestation, and the mean HC/AC, TCD/AC, and erythroblast count were increased. In this study, the TCD is affected in FGR due to uteroplacental insufficiency and, thus, cannot be used to assess GA.

## Conclusion

Using a systematic approach and quantitative evaluation of study methodology, we have provided a review of 8 ultrasound studies of brain structures abnormalities in restricted growth fetuses and have highlighted those of highest quality. High-quality studies were identified for measurement of brain volumes, corpus callosum length, and depth measurements of brain fissures. Nevertheless, all articles provided useful insights on how FGR negatively affects the brain development during fetal life and how fetal brain evaluation is possible and correctly performed by ultrasound techniques. Further research is required with high quality prospective studies to provide more accurate information. In our point of view, it is important to study fetal brain as a whole and not in a compartmentalized way, and the studies should differentiate early-onset from late-onset FGR, once the time in which the insult begins has an impact in which brain structures would be affected. Fetal growth restriction babies are often premature. Brain abnormalities resulting from prematurity, such as cognitive, behavioral, and attentional deficits, as well as major motor deficits (e.g., cerebral palsy), add to those caused by growth restriction; therefore, US biomarkers to identify fetuses with high risk of neurodevelopment impact are paramount in order to trigger strategies such as delivery planning, breast-feeding promotion, and early educational interventions in order to improve neurodevelopment.
